# Controlled Ultrasound-Induced Blood-Brain Barrier Disruption Using Passive Acoustic Emissions Monitoring

**DOI:** 10.1371/journal.pone.0045783

**Published:** 2012-09-24

**Authors:** Costas D. Arvanitis, Margaret S. Livingstone, Natalia Vykhodtseva, Nathan McDannold

**Affiliations:** 1 Department of Radiology, Brigham and Women's Hospital, Harvard Medical School, Boston, Massachusetts, United States of America; 2 Department of Neurobiology, Harvard Medical School, Boston, Massachusetts, United States of America; University of Navarra, Spain

## Abstract

The ability of ultrasonically-induced oscillations of circulating microbubbles to permeabilize vascular barriers such as the blood-brain barrier (BBB) holds great promise for noninvasive targeted drug delivery. A major issue has been a lack of control over the procedure to ensure both safe and effective treatment. Here, we evaluated the use of passively-recorded acoustic emissions as a means to achieve this control. An acoustic emissions monitoring system was constructed and integrated into a clinical transcranial MRI-guided focused ultrasound system. Recordings were analyzed using a spectroscopic method that isolates the acoustic emissions caused by the microbubbles during sonication. This analysis characterized and quantified harmonic oscillations that occur when the BBB is disrupted, and broadband emissions that occur when tissue damage occurs. After validating the system's performance in pilot studies that explored a wide range of exposure levels, the measurements were used to control the ultrasound exposure level during transcranial sonications at 104 volumes over 22 weekly sessions in four macaques. We found that increasing the exposure level until a large harmonic emissions signal was observed was an effective means to ensure BBB disruption without broadband emissions. We had a success rate of 96% in inducing BBB disruption as measured by in contrast-enhanced MRI, and we detected broadband emissions in less than 0.2% of the applied bursts. The magnitude of the harmonic emissions signals was significantly (P<0.001) larger for sonications where BBB disruption was detected, and it correlated with BBB permeabilization as indicated by the magnitude of the MRI signal enhancement after MRI contrast administration (R^2^ = 0.78). Overall, the results indicate that harmonic emissions can be a used to control focused ultrasound-induced BBB disruption. These results are promising for clinical translation of this technology.

## Introduction

Vascular barriers play an important role in the delivery of therapeutics, and can be a significant impediment to effective drug delivery. This is particularly important in the brain, where the blood brain barrier (BBB) excludes most molecules from being delivered from the bloodstream and precludes the use of many drugs [1] for central nervous system (CNS) applications. A number of strategies have been investigated to overcome the BBB, including direct drug injection/infusion [2], trans-arterial infusion of agents such as mannitol to transiently disrupt the BBB [3], [4] or by developing new drug formulations that can cross the BBB [5], [6]. These approaches are either invasive, not targeted, or require the development of novel drugs or drug carriers.

A promising noninvasive approach to deliver drugs past the BBB is the use of focused ultrasound with microbubbles, which can induce targeted BBB disruption for a few hours and allow drugs to be delivered to the brain [7]. This method utilizes mechanical interactions between the microbubbles oscillating in the ultrasound field and the vasculature, leading to a transient disassembly of tight junction complexes and the induction of active transport [8], [9]. If this approach can be scaled up to human use and effectively controlled, it could have a large impact on CNS therapeutics.

Past work has identified a relatively narrow window in acoustic pressure amplitude where BBB disruption can be safely achieved [10], [11]. Without adequate control of the sonications, the ultrasound exposures (sonications) can create excessive forces in proximity to the oscillating microbubbles, leading to vascular damage [12], or in very small oscillations, leading to insufficient local perturbation and lack of the desired effect [10]. Moreover, it is difficult in practice to precisely predict (e.g. within the safety window) the acoustic pressure amplitude produced by any administered ultrasound acoustic power in vivo, particularly when sonicating transcranially. Vascularity, vessel diameter, blood flow and other properties also vary substantially across different structures of the brain, which can impact the local concentration of microbubbles, how they interact with the ultrasound field, and how much drug will be delivered to the brain [13]. These uncertainties, along with the nonlinear response of microbubbles [14], [15], makes control critical for the utilization and clinical translation of this technique.

The acoustic emissions from the oscillating microbubbles offer characteristic signatures that allow for remote assessment of the mode of oscillations [16] and offer a potential way to guide and monitor microbubble-enhanced ultrasound therapies such as BBB disruption [10], [11], [17], [18]. The spectral content and strength of the emissions can be used to monitor the micro-scale perturbations. In particular, microbubbles vibrating in an ultrasound field (“stable cavitation”) can exert direct forces on the endothelium through oscillatory and radiation forces. They also can exert indirect shear forces [18], [19] induced by micro-streaming [20] in the fluid that surrounds them. Presumably these forces produced during stable cavitation are responsible for the observed BBB disruption [10], [11].

Strong harmonic and/or sub- and ultra-harmonic acoustic emissions in the absence of broadband signal are indicative of such stable volumetric oscillations [21], [22]. At higher pressure amplitudes the microbubble oscillations deviate significantly from the equilibrium radius and become unstable. At a high enough pressure amplitude, the microbubble can collapse violently due to inertia of the surrounding medium, which can produce large shear stresses, shock waves [23], elevated temperatures [24], and, when the collapse happens in proximity to interfaces (e.g. vascular walls), micro-jets [25], [26] and membrane perforation [12], [27]. This collapse, termed “inertial cavitation” [28], creates a pressure spike [29] that is manifested in the frequency domain of the acoustic emission as a broadband signal. Inertial cavitation has been associated with tissue damage [30].

The purpose of this work was to integrate an acoustic emissions monitoring system into a clinical transcranial MRI-guided focused ultrasound (TcMRgFUS) system and to evaluate its use for controlling BBB disruption in non-human primates. The system uses harmonic and broadband emissions, signatures of the effectiveness of the ultrasound to disrupt the BBB and of tissue damage, respectively [10]. We have utilized a spectroscopic method for monitoring the acoustic emissions [31] that largely isolates the emissions arising from microbubble activity. This analysis, along with the design of the monitoring system, aimed to maximize its sensitivity to the harmonic and broadband emission signals, which are small compared to the fundamental frequency of the TcMRgFUS device, particularly when sonicating transcranially. The system was characterized in pilot studies over a wide range of exposure levels. It was then used during tests evaluating the safety of repeated BBB disruption sessions in macaques [32], where it was used to control the procedure. Here, we report on the success of this control, which aimed to reliably induce MRI-detectable BBB disruption without the production of broadband emissions. We also evaluated whether the strength of the harmonics emissions was predictive of whether or not BBB disruption was produced, and if its strength could predict its magnitude. Finally, we explored strategies to increase the strength of the harmonic emissions, and presumably the magnitude of the BBB disruption. These experiments, in which the operator used the acoustic emissions analysis to manually adjust the exposure level at each target in each animal, aimed to investigate whether this system and analysis can form a basis for the future development of an automated, computer-based real-time controller.

## Materials and Methods

### Ultrasound Device

**Figure 1:**
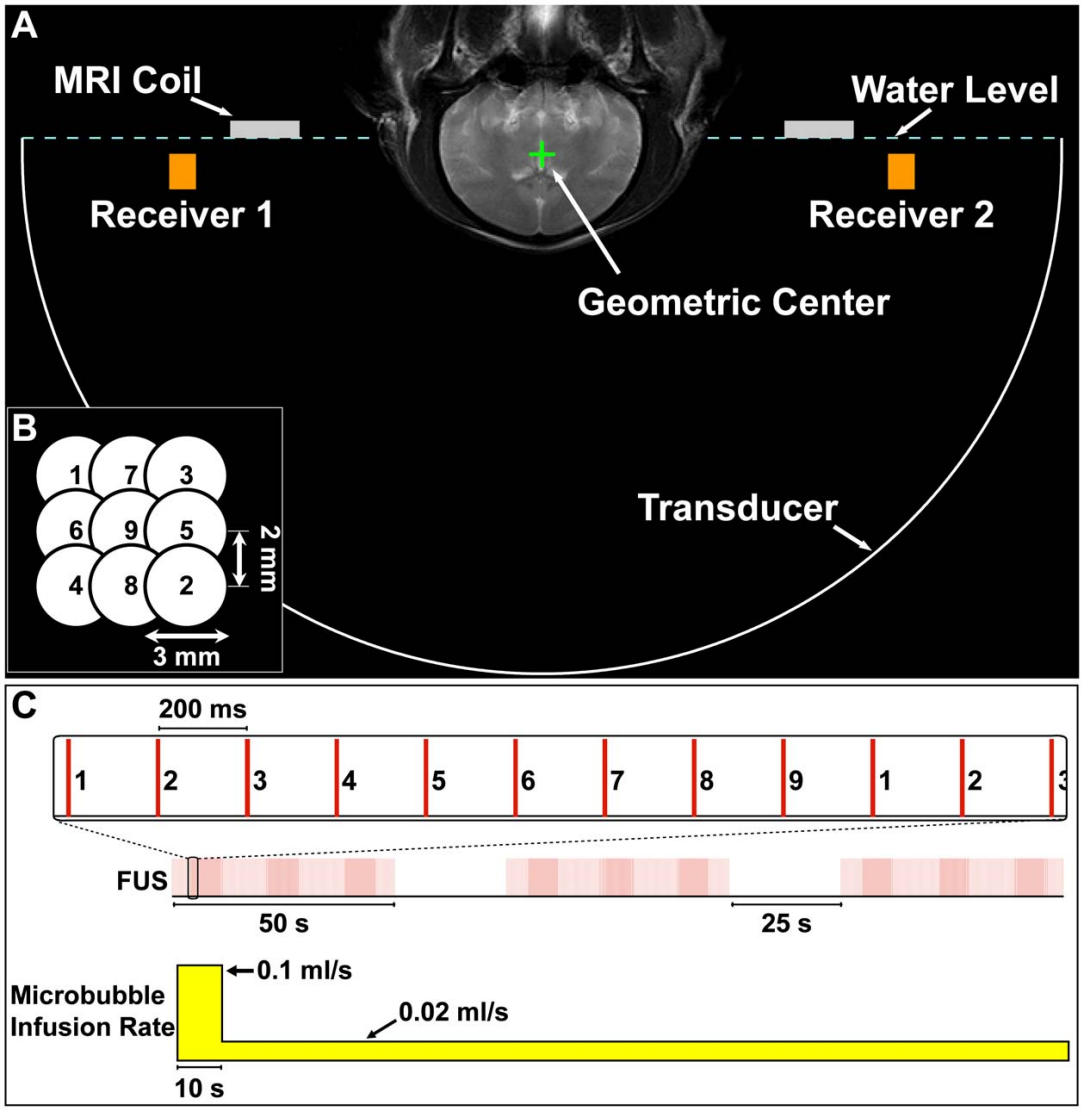
Experimental setup and methods. (A) Coronal T2-weighted MRI of a monkey obtained during one of the experiments. The image has been annotated to show the location of the 30 cm diameter hemisphere transducer, the two transducers that served as receivers to monitor the acoustic emissions, and the MRI surface coil. The annotations were drawn to scale with the location of the brain in a typical position. (B) Beam steering pattern used during the multi-target sonications. The order of the sonications delivered is indicated. (C) Pulsing scheme used during the multi-target sonications. Each 10 ms burst was applied in sequence to the different subsonication targets every 200 ms. The pattern was repeated every 1.8 s, resulting in a pulse repetition frequency at each target of 0.55 Hz. Three 50 s sonications were delivered in series using this pattern, with a 25 second delay between sonications. The microbubbles were administered as an infusion that was started at the beginning of the each multi-target sonication, as indicated. This infusion was delivered at a variable rate in order to quickly reach a steady-state microbubble concentration in the tissue and maintain it throughout the entire sonication.

The ultrasound fields were generated by a clinical TcMRgFUS system (ExAblate 4000 low frequency, InSightec Ltd, Haifa, Israel) originally developed for high-intensity sonications for tissue ablation [33]. This system uses a phased array with 1024 elements arranged in a 30 cm diameter hemisphere with a central frequency of 220 kHz. It was operated in burst mode via a gating signal provided by an arbitrary waveform generator (model 396, Fluke, Norwich, UK), which also triggered the acquisition for the system used to monitor acoustic emissions. The TcMRgFUS system was integrated with a clinical 3T MRI unit (GE Healthcare, Milwaukee, WI). Imaging was performed using a 15 cm diameter surface coil (constructed in house). The TcMRgFUS array faced upwards (i.e. rotated 90° from its normal use in patients [34]) and was filled with degassed water. The animal was placed supine on the MRI scanner table with its head tilted backwards so that the top of the head was submerged in water (Fig. 1A).

The driving system of the TcMRgFUS system allows for individual control of the phase and amplitude for each element in the phased array so the beam can be steered several cm in each direction, enabling targeting of different brain regions without moving the transducer. The steering range of the transducer was sufficient to cover the entire brain in a monkey. During the experiments the beam can be steered to different targets during a single sonication. In this way multiple “subsonications” can be delivered in sequence to multiple locations in a single sonication. The acoustic power can be set individually for each of these subsonications. The phased array is also used to correct for skull-induced beam aberrations [35]. These corrections were not performed in these experiments, as they use modeling based on CT scans of the skull, which were not available to us at the time of these experiments. Note however that only limited beam aberration is expected at this frequency (220 kHz) [36].

The half-intensity profile of the focal region in water was provided by the manufacturer and in the lateral and axial directions were approximately 3.0 and 5.8 mm, respectively. Reported values for the ultrasound exposure levels are in vivo estimates of the peak negative pressure amplitude (referred throughout as simply “pressure amplitude”). To estimate the in vivo pressure amplitude, measurements were first obtained in water in the free field as a function of the acoustic power using a 4 mm diameter, calibrated, omni-directional hydrophone (TC 4038, Reson Inc, Slangerup, Denmark). To estimate the effects of a monkey’s skull on the pressure amplitude, we degassed a desiccated rhesus macaque skull in water for several days. The insertion loss due to this skull was measured at multiple positions with this hydrophone and a single-element FUS transducer (diameter/radius of curvature: 10/8 cm) operating at 257 kHz. The drop in pressure amplitude due to the monkey skull was 25±14%. Attenuation from brain tissue and skin were not considered, as their impact would be less than 5% at 220 kHz. Based on these measurements, an acoustic power level of 1 W was estimated to produce a pressure amplitude of 223 kPa in the brain. Other pressure amplitudes were estimated by extrapolation assuming linear propagation. Note that this pressure amplitude estimate did not include effects arising from variations in skull bone thickness for different animals, variability in skull orientation within the TcMRgFUS system, or decreases in pressure amplitude that occur when the focal point is steered electronically away from the geometric focus. These effects were expected to contribute uncertainty to our pressure amplitude estimates. The presence of standing waves may have added additional uncertainty. While we have not observed evidence of significant standing waves such as BBB disruption in the beam path [32], they may have been present with this device at a low level [37], [38].

### Sonications

Similar to prior work [7], the sonications consisted of 10 ms bursts applied at a low pulse repetition frequency (PRF). For sonications at individual targets (i.e., without beam steering during sonication) in our pilot studies, a 1 Hz PRF was used. For multi-target sonications, the beam was steered sequentially to nine subsonication targets arranged in a 3×3 grid in a single plane with a 200 ms interval (Fig. 1B). This interval was the fastest that the FUS device could be programmed to sonicate different targets in sequence, which reduced the duty cycle per subsonication target. The pattern was repeated every 1.8 s, yielding a PRF at each location of 0.55 Hz. Three 50 s sonications were delivered in sequence with a delay between sonications of ∼25 s (Fig. 1C). This delay was imposed by the TcMRgFUS system software, which limited the sonication duration to 50 s when such multi-target sonications were employed. The subsonications were set 2 mm apart with an aim of creating a volume of BBB disruption of approximately 1 cm^3^.

Except where specified, a single acoustic power level was used during each burst and subsonication target during each multi-target sonication. The power level used varied for the different animals and brain structures targeted in each animal and was set based on online measurements of the acoustic emissions, as we describe below. Before any microbubbles were administered, each target was sonicated for 25 s without microbubbles using identical parameters. These “baseline” sonications were used in the acoustic emissions analysis, as described below.

For BBB disruption, each sonication was combined with an infusion of microbubble ultrasound contrast agent. The microbubble agent Definity (Lantheus Medical Imaging, N. Billerica, MA) was infused over the entire sonication via an MRI-compatible infusion pump (Spectra Solaris EP, Medrad, Warrendale, PA). The microbubble agent was diluted in 5 ml sterile phosphate-buffered saline. The infusion was administered at a variable rate. The first 1 ml was administered at 0.1ml/s for 10 s. The remaining 4 ml was infused at a slower rate of 0.02 ml/s for 200 s (Fig. 1C). This infusion protocol was employed in order to rapidly reach a steady-state tissue concentration of microbubbles, and then maintain it throughout the entire sonication. The infusion started simultaneous with the sonication, which enabled us to observe the change in acoustic emissions when the microbubbles arrived at the focal region. Except where specified, a dose of 20 µl/kg of Definity was used for each infusion. The time between sonications at different locations within the brain was typically 2 min. This time allowed most of the microbubbles to be cleared from the vasculature.

### Acoustic Emissions Monitoring System

The acoustic emissions were recorded for every 10 ms burst (Fig. 1C) with two MRI-compatible piezoelectric transducers, which were constructed in-house for this study. Since the skull attenuates high ultrasound frequencies, receive transducers sensitive at frequencies below 1 MHz were selected for recording the emissions. We aimed to have maximum sensitivity to broadband emissions, which can be smaller in magnitude than the fundamental frequency or harmonics of the TcMRgFUS device during BBB disruption [10]. This sensitivity was achieved using filtration to reduce the fundamental frequency and through the use of sharply-tuned receive transducers with a resonant frequency of approximately 610±20 kHz. This frequency lies between the third harmonic (660 kHz) and the fifth ultraharmonic (550 kHz) of the TcMRgFUS device. The two transducers were rectangular, air-backed, and weakly focused (radius of curvature: 15 cm). The piezoelectric element of each transducer was made of lead zirconate titanate and had dimensions of 7×40 mm. The −3 dB of the sensitivity profile of the transducers (measured at 610 kHz with a needle hydrophone) were 100, 24, and 6 mm in the axial and the two transverse dimensions, respectively, with maximum sensitivity at 75 mm away from the transducer face. Each transducer was mounted in an acrylic housing (dimensions: 5×2×1 cm). The transducers were mounted in the water in the beam path of the TcMRgFUS device on each side of the head, approximately 10 cm from the geometrical focus of the hemispherical phased array (Fig. 1A). Their effect on the beam path was assumed to be negligible. At 10 cm from the focal point, the FUS beam transverses a hemisphere with a surface area of 628 cm^2^. With a cross-sectional area in the beam path of 5 cm^2^ each, the transducers blocked less than 2% of the transmitted field, therefore their effect on the beam should be minor. The transducers were connected to the data acquisition system through the penetration panel of the MRI room with approximately 10 m coaxial cables.

Two filtering/amplification schemes were evaluated. One transducer was connected to a 20 dB gain low-noise preamplifier and a 250–1000 kHz band-pass filter (EC 6081, Reson Inc, Slangerup, Denmark). The other was connected to a 125–390 kHz band-reject filter with a 40 dB gain (Model 3944, Krohn-Hite Corp, Brockton, MA, USA). The signals were recorded using a high-speed digitizing card (NI PXI-5124 National Instruments, Austin, Texas, USA) that had 512 MB onboard memory per channel, 12 bit resolution, and a maximum sampling rate of 200 Ms/s. The digitizer was driven by an 8 core, 2.53 GHz PC with 12 GB memory (Dell Precision T7500, Round Rock, Texas, USA) and was able to transfer the data at a speed of 800 MB/s. The system was controlled using software developed in-house in Matlab (Mathworks, Natick, MA, USA).

The voltage traces measured by the receive transducers from the entire 10 ms burst were recorded for every sonication. The data was digitized at a Nyquist frequency of 5 MHz, well above the frequency components of the recorded emissions and the sensitivity of our recording transducers. The spectral resolution was 100 Hz. The control software displayed both time and spectral data, which was obtained via fast-Fourier transform (FFT), from both detectors in real-time. To decrease spectral leakage, a Hanning window was applied to the time-series data before computing the FFT. MRI was not performed during the acoustic emissions acquisitions to avoid artifacts induced by the scanner.

### Acoustic Emission Analysis

The central concept has been to develop a spectroscopic approach to evaluate the microbubbles’ emissions while minimizing the influence of background signals arising from linear and nonlinear components of the transmitted and reflected acoustic wave produced by the TcMRgFUS device and from background electronic noise. During the sonications the acoustic emissions captured with the two transducers are a mixture of many sources that need to be decoupled from activity at BBB disruption site, presumably at the focal region. This was achieved by taking the ratio of the acoustic emissions with microbubbles to that obtained during identical sonications without microbubbles.

To obtain the spectral decomposition needed to evaluate microbubble response in different frequency bands, the power spectral density (PSD) of the digitized RF signals recorded from the passive cavitation detectors was calculated. The PSD of a discrete time series of data 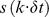 is expressed in units of V^2^Hz^−1^ and is given by:
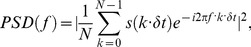
(1)Where 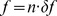 is the discrete frequency interval *n* in which the PSD is evaluated, 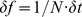 is the spectral resolution (100 Hz here), 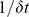 is the sampling frequency (1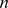 here), and 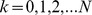, where *N* is the time-series length (10^5^ here). The PSD measured from the acoustic emissions during the sonications incorporates microbubble emissions, the transmitted wave from the FUS transducer, from reflections of this wave from the tissue, and harmonic waves due to nonlinear sound propagation. Electronic noise from surrounding equipment will also be present. The recorded signal is modulated by the frequency response of the transducers. All these components confound the analysis of the power spectrum and interfere with accurate assessment and characterization of the microbubble oscillations.

In order to separate these sources from the microbubble emissions, we obtained background acoustic emissions at every target in identical sonications applied before any sonication with microbubbles. We then determined the relative power spectral density (RPSD) [31]:
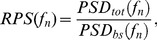
(2)where 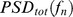 is the total recorded energy per frequency bin during the sonication, 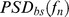 is the recorded power per frequency bin in the absence of microbubbles (*bs* stands for “baseline” signal). The relative signal strengths of the harmonic and ultraharmonic emissions can be determined from the log transformed RPSD. Reported values are the mean of the first three harmonics (440, 660, and 880 kHz) and ultra-harmonics (330, 550, and 770 kHz). For broadband emissions, a frequency band around the resonance frequency of the receiving transducers (610 kHz) was used. A log transform was performed to simplify the statistical analyses; otherwise the emission measurements were not normally distributed. The strength of the acoustic emissions is summarized in the following equation
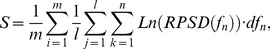
(3)where 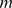 is the number of waveforms that were averaged together (typically 75 for harmonics; 1 for ultraharmonic and broadband signals), 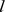 is the number of spectral bands analyzed (3 for harmonic/ultraharmonic peaks, one for broadband signal), and 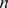 is the number of discrete frequency bands used for each measurement. For harmonic and ultraharmonic emissions, 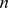 was five, which corresponded to five points in the discretized spectrum that covered a frequency band of about ±250 Hz. For broadband emissions, 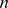 was 100, which corresponded to a frequency band of ±5 kHz. The units of 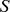 are 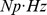.

The emissions produced during sonication with microbubbles are likely to be small and will be attenuated by the skull. It is therefore important to determine whether the measurement at each frequency band is significantly above the noise of the RPSD. Thus, in addition to calculating the relative signals (harmonics, ultraharmonics, broadband), we also calculated the signal-to-noise ratio (SNR). The noise for each measurement was
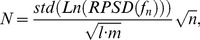
(4)where 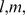 and 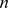 are defined in Eq. 3. It was evaluated at 1150 kHz in the same way as the broadband noise. This was a region of the spectrum that no signal related to microbubble emission was observed. We used a conservative SNR of 3 to classify an emission as significantly above the noise floor.

In processing the recordings, we treated the measurements from the two receiving transducers as equivalent; reported values for each sonication are from the transducer that had the larger signal. This was possible because despite using different filtration schemes, the measurements for the two transducers were found to be correlated, and the measurements on average were the same. Linear regression of the harmonic emissions signal strength (defined above) for the two transducers showed a good correlation (R^2^: 0.65), and the two signals were found to be not significantly different (P<0.05) using a paired t-test.

The use of an infusion for the microbubble administration resulted in a harmonic signal that was steady over time over most of the sonication and enabled us to use an ensemble of spectral data in Eq. 3 to increase accuracy in harmonic signal measurements per location. During the multi-target sonications, typically the last 75 waveforms were averaged together for each subsonication target. When broadband or ultraharmonic emissions were observed, they were often sporadic and variable in magnitude, so instead of averaging the data, the maximum relative broadband signal of all waveforms were computed.

### Animals

All experiments were done in accordance with procedures approved by the Harvard Medical School Institutional Animal Care and Use Committee. The animals were anesthetized during all the procedures and were constantly monitored throughout and after recovery. No pain or suffering was evident as a result of the procedures. Monkeys were housed, fed, watered, socially housed, and provided with environmental enrichment according to U.S. Department of Agriculture (USDA), Office of Laboratory Animal Welfare (OLAW), and Association for Assessment and Accreditation of Laboratory Care (AAALAC) regulations.

Acoustic emission measurements were obtained in six macaques. Pilot studies to explore a large range of power levels were performed on monkeys #1–2. Monkey #2 was euthanized after the experiments, and the sonicated locations were examined in histology, as described below. The sonications in monkeys #3–6 were part of a survival study on the safety of repeated BBB disruption [32] (see below for details).

Monkeys #1–5 were adult rhesus macaques (three male, one female, weight: 7–13 kg); monkey #6 was a juvenile nemestrina macaque (male, 3.75 kg). Each animal was anesthetized with ketamine (15 mg/kg/h i.m.) and xylazine (0.5 mg/kg/h i.m.), or with 4 mg/kg/h ketamine and Dexmeditomidine (0.01–0.02 mg/kg/h i.m.) and intubated. The head was shaved, and a catheter was placed in a leg vein. During the procedure the heart rate, blood oxygenation levels, and rectal temperature were monitored. Body temperature was maintained with a heated water blanket.

### MR Imaging and Analysis

MRI was performed before the animal experiments to localize the focus of the TcMRgFUS device in the MRI image space. During the experiments it was used for treatment planning to select the brain targets and after treatment to assess the treatment (BBB disruption and tissue damage). During the sonications, no MRI was performed; the treatment was controlled solely using acoustic emissions as described above.

Before each experiment, the location of the ultrasound beam in the MRI coordinate-space was found by visualizing focal heating in an FUS/MRI phantom using MR temperature imaging [39]. Then the animal was placed on the FUS system, and MRI was used in order to select identify the different brain targets for sonication. We used a 3D fast spoiled gradient echo sequence with inversion recovery preparation (TR/TE/TI: 5.3/2.0/600 ms, FA: 10°, FOV: 12 cm, matrix: 128×128, slice thickness: 2 mm) or a multi-slice T2-weighted Fast Spin Echo (FSE) sequence (TR/TE: 4500/85.8 ms; echo train length, ETL: 8; field of view, FOV: 12 cm; matrix: 256×256, slice thickness: 3 mm) for this planning. Different targets were selected with the aid of an MRI atlas of the rhesus macaque brain [40].

At the end of each session (a few minutes after the last sonication), we acquired T1-weighted FSE images (TR/TE: 500/14 ms; ETL: 4; FOV: 12 cm; matrix: 256×256, slice thickness: 3 mm). These images were repeated after the administration of the MRI contrast agent Gd-DTPA (Magnevist, Berlex Laboratories, Inc., Wayne NJ) at a concentration of 0.1 mmol/kg of body weight as a bolus injection through the leg vein. This contrast agent normally does not extravasate into the brain, and signal enhancement after Gd-DTPA injection was used to identify regions of BBB disruption. A 3D T2*-weighted spoiled gradient echo sequence (TR/TE: 33/19 ms; FA: 15°; FOV: 12 cm; matrix: 256×256; slice thickness: 1 mm) was used to detect vascular damage. This sequence shows hypointense regions induced by tiny red blood cell extravasations (petechaie) that occur presumably due to inertial cavitation [7]. T2-weighted FSE imaging was also acquired after sonication.

The contrast-enhanced T1-weighted images were scored as enhancing or not by an author who was blind to the acoustic emissions analysis. This author also compared the T2*-weighted images acquired before and after sonication and scored each targeted region as having or not having hypointense spots. Images and plots of MRI contrast enhancement show the percent signal enhancement relative to pre-contrast imaging.

### Experimental Protocols

#### Pilot studies

Experiments were performed in monkeys #1–2 to characterize the acoustic emissions monitoring system over a relatively wide range of exposure levels, including those that produced significant broadband emission, a signature for inertial cavitation. We aimed to verify that the system functioned as expected based on prior work in small animals. We aimed to verify that the harmonic emissions occurred at a lower pressure amplitude than broadband emissions and to confirm that the system could detect low-level broadband emissions which have been correlated with the production of minor vascular damage (petechaie) [10], [11]. To minimize the amount of brain damage induced by the exposures, single-target sonications were performed during these tests.

In monkey #1, we evaluated the acoustic emissions as a function of the peak negative pressure amplitude. During these sonications, the acoustic power increased with every burst (between 0.4 and 4 W in 10 steps). This range corresponded to estimated pressure amplitudes in the brain of 140–440 kPa. This cycle was repeated 4 times for each sonication. We then identified the threshold for harmonic, ultraharmonic, and broadband emissions with an SNR>3. Four targets were sonicated with this scheme in the amygdala in this animal. This structure was targeted in order to establish applicability for deep brain targets.

Tests were performed in monkey #2 to investigate the sensitivity of our detectors to low-level broadband emissions. Here we sonicated at 10 targets in the cingulate cortex at different pressure amplitudes. Five different exposure levels were tested between 0.3 and 1.5 W (estimated pressure amplitude in the brain: 125, 175, 210, 245 and 275 kPa); each exposure level was tested at two targets. The cingulate cortex was selected because it is an anatomically large and homogeneous gray matter target that is aligned with the axial MRI planes. This monkey was sacrificed approximately two hours after the last sonication for histological examination. The animal was deeply anesthetized with ketamine (15 mg/kg i.m.), given an overdose of pentothal (100 mg/kg), and then perfused transcardially with 1 L 0.9% NaCl, followed by 2 L 10% buffered formalin phosphate). The brain was removed and bisected midsagitally, cut into approximately 4 mm thick axial slabs, and photographed. The sonicated regions were identified and extracted from these slabs into 2×2 cm blocks, and then cut into a series of 5-µm-thick paraffin-embedded sections. Every 40th section was stained with hematoxylin and eosin (H&E) and Nissl to evaluate whether extravasated red blood cells (petechaie) or other tissue damage were present.

#### Acoustic emissions-based control

Monkeys #3–6 are part of an ongoing survival study where repeated BBB disruption was produced in targets in the visual system followed by functional/behavioral tests [32]. In each animal, the lateral geniculate nucleus (LGN) and the foveal confluence of primary visual cortex and secondary visual areas were sonicated in both hemispheres in five weekly sessions. Additional locations centered on the cingulate cortex which included adjacent white matter were targeted specifically for the present study (see below for details). Each target in these animals utilized multi-target sonications, and every sonicated volume included both gray and white matter structures. Targets in the visual cortex also often included sulci, and some overlapped the brain surface.

The acoustic emissions were used to control the acoustic power level at the different targets in each animal. Strong harmonic emissions were tested as a signature for BBB disruption, and broadband emissions were considered signatures for overexposure and a risk for vessel damage. The control was performed manually. In the first session in each animal a conservative power level was used, which was selected based on our estimates for the pressure amplitude in the monkey brain described above, prior work in small animals that evaluated BBB disruption thresholds [41], and our experience in earlier sessions. If at this initial power level we did not observe an increase in harmonic emissions in at one or more of the subsonication targets that was at least one to two orders of magnitude larger than the baseline emission obtained earlier without microbubbles, the power was increased and the sonication repeated. This procedure was repeated at each target in each animal. Over the following weeks, this power level was used as a starting point, with minor week-to-week increases or reductions employed if later detailed offline analysis revealed weak harmonic emissions and/or contrast enhanced MR signal enhancement or broadband signal was detected. Overall, 114 volumes were sonicated over the course of these 22 experiments in monkeys #3–6. The acoustic power level ranged from 0.2–1.9 W, which yielded an estimated pressure amplitude in the brain 100–300 kPa. The applied acoustic power varied among the different brain targets and animals (Table 1), with larger animals generally requiring higher levels to achieve strong harmonic emissions.

**Table 1:** Acoustic power level used at the different targeted structures in each animal.

Monkey	Weekly Sessions	Sex, weight	LGN	Visual Cortex	Cingulate Cortex	Other
1	1	M 5 kg	–	–		0.4–4 W (N:4)
2	1	M, 7.5 kg	–	–	0.3–1.5 W (N:10)	
3	5	M, 7 kg	0.81 [0.75–0.90] W^1^ (N: 10)	0.65 [0.50–0.75] W (N: 6^2^)	–	–
4	5	F, 7.5 kg	0.88 [0.65–1.20] W (N: 10)	0.68 [0.65–0.70] W (N: 10)	0.55 [0.20–0.85] W (N: 8)	0.70 [0.70–0.70] W (N: 2)
5	5	M, 13 kg	1.58 [0.85–1.90] W (N: 10)	0.63 [0.60–0.66] W (N: 9^3^)	–	1.59 [0.85–1.90] W (N: 9)
6	7	M, 3.75 kg	0.54 [0.50–0.60] W (N: 10)	0.37 [0.35–0.40] W (N: 10)	0.47 [0.41–0.55] W (N: 20)	–

^1^Mean power of N sonications [range]. The pressure amplitude (Pa) estimates in the brain in kPa were found using the following relationship: Pa = 223*(Power)^1/2^.

^2^The visual cortex targets were not sonicated in the first session with this animal.

^3^Acoustic emissions data excluded from one sonication due to excessive electronic noise.

To test how well this control worked in ensuring BBB disruption without producing broadband emission, we counted the number of multi-target sonications that resulted in evident contrast enhancement in MRI after Gd-DTPA administration in at least one subsonication target in the LGN and visual cortex sonications. These two structures were included in this analysis as we always aimed to produce BBB disruption in them (some cingulate cortex and other targets used lower power levels to evaluate the acoustic emissions below the BBB disruption threshold). We also counted the number of subsonications and individual 10 ms bursts where broadband emissions with an SNR greater than 3 were evident.

### Acoustic Emissions vs. MRI

After the experiments in monkeys #3–6, the acoustic emissions data was compiled and compared retrospectively to MRI exams obtained immediately after the sonications. First, we examined whether the strength of the harmonic emissions was predictive of the onset for BBB disruption. This examination was performed on all of the targets sonicated in monkeys #3–6. BBB disruption at each target was ascertained by the presence or absence of signal enhancement in T1-weighted MRI after the injection of Gd-DTPA. The binary (Yes/No) outcome of the contrast enhanced MR image assessment was compared to the strength of the harmonic signals recorded during the sonications. The subsonication with the maximum harmonic emissions signal was used for this analysis.

Next, we investigated whether the strength of the harmonic emissions was predictive of the level of the BBB disruption. Here, we aimed to correlate the emissions signals for individual subsonication targets to the corresponding signal enhancement after Gd-DTPA administration. Due to the three-dimensional complexity of the gray and white matter structures in the visual cortex and LGN, we were unable to consistently identify which enhancing spot in MRI corresponded to which subsonication target in these two structures. This discrimination was confounded by leakage of contrast agent from one target to another and one imaging plane to another (particularly when the contrast leaked into the sulci), and small shifts of a few mm in the position of the head over the course of the experiments. While this discrimination was possible in a few cases, in most cases we were not confident in our ability to associate the enhancement with particular subsonications. In the cingulate cortex in contrast, this discrimination was relatively straightforward since the orientation of the cortical structure was parallel to our imaging planes. Thus, in the 28 cingulate cortex targets, we were able to make this comparison. However, we were not able to make this comparison for all subsonication targets due to evident leakage of MRI contrast agent between the targets. Thus, we compared MRI signal enhancement at the subsonication with the biggest enhancement to the corresponding harmonic emissions signal.

Finally, we examined the T2*-weighted imaging obtained after each session and identified whether or not hypointense spots were produced at any of the subsonication targets, and whether they correlated with the presence of broadband or ultraharmonic emissions. This identification was often challenging, as the signal changes induced with minor petechaie can be subtle; this procedure was facilitated by registering the images to those obtained in other sessions [32].

### Sonication Optimization

Finally, we evaluated the feasibility of increasing the harmonic emissions, and presumably the BBB disruption, in individual subsonication targets where low signals were recorded. In these tests, a multi-target sonication was applied at a nominal exposure level as described above. We then analyzed the acoustic emissions (Eq. 3) for each individual subsonication target to determine which had no or only weak harmonic emissions signals. Those targets were sonicated a second time with either a small increase in power (corresponding to a pressure amplitude increase of 5–15 kPa), or at the same power level but with five times the microbubble dose. Subsonication targets that exhibited strong harmonic emissions were not sonicated again. These experiments were performed over several weekly sessions in monkeys #5–6. The procedures were evaluated in 11 multi-target sonications in the cingulate cortex, four of which at higher microbubble dose, and one in the visual cortex. The sub-sonication targets were selected for a second sonication solely based on the harmonic signal and not based on the underlying structure.

We aimed with these experiments to determine whether additional sonications could increase the harmonic emissions signal above a threshold value where MRI contrast enhancement was expected, based on the experiments described above. We also compared the harmonic emissions signal strength for the first and second sonications to determine whether any increase that we produced was predictable. For experiments where the acoustic power was increased, we compared our results with our pilot study where a wide range of exposures were delivered in sequence to individual targets. For experiments that increased the dose of micro-bubbles, we investigated whether the harmonic emissions signal strength would scale with the microbubble dosage.

### Statistical Analysis

Each power spectrum obtained in the presence of microbubbles was divided by the average spectrum obtained during 25 s sonications that were performed before any microbubbles were injected (10 or more waveforms per subsonication location) according to Eq. 2. Harmonic emissions signals were relatively constant over most of the sonications. Reported harmonic emissions signals are the average microbubbles at the sonicated targets are reported (typically the last 75 measurements). Reported broadband and ultraharmonic signals are the maximum individual measurement achieved among all the waveforms for that sonication. The harmonic emission signals for different tissue structures were compared using an unpaired, two-tailed t-test. Additional analysis included least-squares regression and calculation of correlation coefficients.

## Results

### Pilot Studies

**Figure 2:**
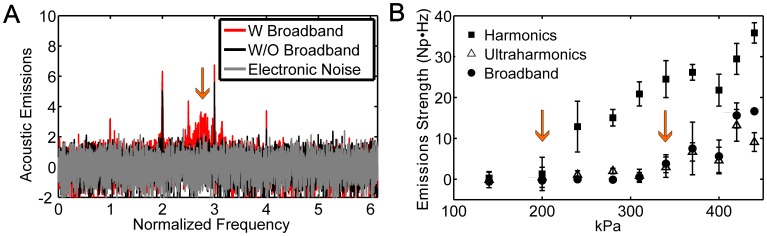
Acoustic emissions over a wide range of exposure levels. (A) Typical power spectra showing spectra with and without broadband emission. The emissions recorded during sonication with microbubbles were normalized to baseline data obtained during identical sonications without microbubbles according to Eq. 2. Wideband emission was observed as signal detected around 610 kHz (arrow), the resonant frequency of our receiving transducers. (B) Mean acoustic emissions signal (± S.D.) as a function of the estimated pressure amplitude in the brain for harmonic, ultraharmonic and broadband emissions obtained in monkey #1 during a sonication where bursts were applied sequentially at increasing pressure amplitudes. Between 200–400 kPa, the harmonic signal strength increased linearly as a function of pressure amplitude (R^2^ = 0.92). The harmonic signal increase per kPa was 0.14±0.01 Np·Hz/kPa. The average signals from four bursts at each pressure amplitude are shown (mean ± SD shown). The arrows indicate the lowest pressures where the harmonic and broadband signals were observed with an SNR greater than 3. Broadband and ultraharmonic emissions were only observed in these locations when the harmonic emissions were greater than 20 Np·Hz.

The acoustic emissions system was evaluated in initial, acute tests in monkeys #1–2, where a range of pressure amplitudes were tested and included exposures that purposely induced broadband emissions. Fig. 2A shows typical acoustic emissions spectra for cases with and without broadband emissions. Broadband emissions were observed as signal at the resonant frequency of the narrowband receiver transducers and were generally at substantially lower amplitude than the harmonic emissions. Note also the flat frequency response of the recordings and the suppression of the large signal at 220 kHz after normalizing the data to recordings made earlier without microbubbles. Fig. 2B shows the increase in signal for different frequency bands as a function of the estimated pressure amplitude in the brain. A marked increase in harmonic emissions was observed starting at around 250 kPa, which increased linearly as the pressure amplitude was increased at a rate of 0.14±0.01 per kPa. Ultraharmonic (signal at 3/2 and 5/2 of the ultrasound frequency) and broadband emissions occurred at higher pressure amplitudes than for harmonic emissions, and their onset occurred at similar thresholds (340 kPa) for this target.

**Figure 3:**
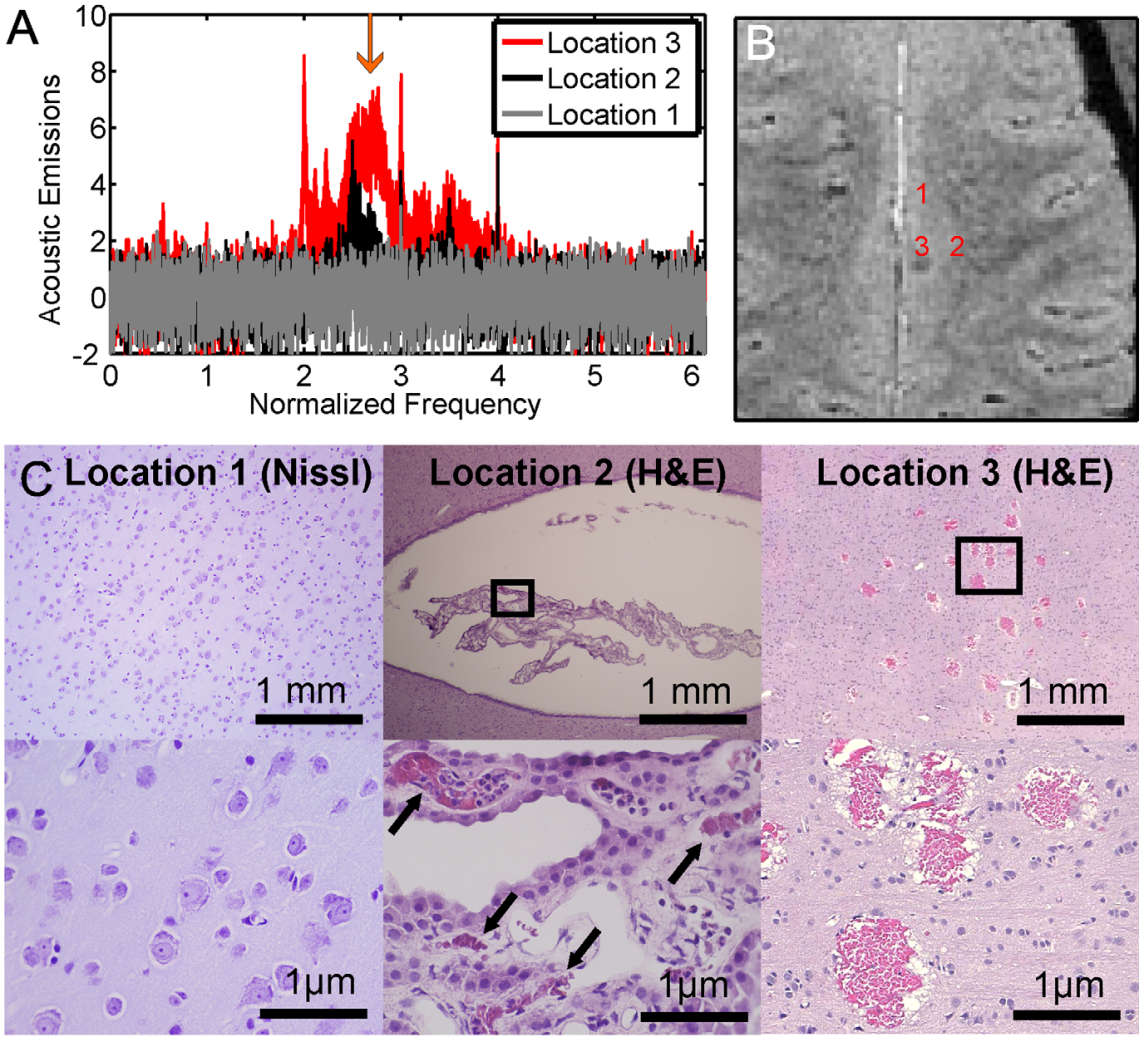
Acoustic emissions, MRI, and histology at different exposure levels. (A) Acoustic emissions, recorded during three single-target sonications delivered to the cingulate cortex in monkey #2, showing the largest broadband emissions for these sonications (arrow). Only locations 2 and 3 exhibited strong harmonic emissions (harmonic signal strength: 4.1, 17.9 and 25.8 Np·Hz, for locations 1–3, respectively) and resulted in contrast enhancement in MRI. Broadband emissions with an SNR greater than 3 were present at location 3 and at a much lower level at location 2, but not at location 1. The estimated pressure amplitudes in the brain were 175 kPa at location 1 and 275 kPa at locations 2–3. (B) T2*-weighted image acquired shortly after the sonications. A hypointense spot is only evident at location 3. (C) Light microscopy showing no petechaie or other changes at location 1. Tiny petechaie were found in the choroid plexus, (in the lateral ventricle) which was just inferior to location 2. It is possible that any changes in MRI resulting from petechaie at this location were missed because they occurred in the ventricle, which appears hypointense in our T2*-weighted imaging. Extensive petechaie were evident in histology at location 3. These petechaie covered a region 3 mm in diameter, similar to the half-intensity beam width of the focal zone of this TcMRgFUS device.

Sonications were performed in monkey #2 to assess the sensitivity of the system in detecting low-level broadband emissions. Fig. 3 shows the results of this experiment. Of the ten locations targeted, three were found to be enhancing in MRI with contrast and had strong harmonic emissions signals. Two of these targets exhibited broadband emissions with an SNR greater than 3 (Fig. 3A), with a hypointense spot evident in T2*-weighted imaging detected for the location with the stronger emissions (Fig. 3B). This location resulted in extensive petechaie in histology (Fig. 3C). The location with the lower broadband emissions signals, which were near our sensitivity limits (SNR = 4.3), resulted in a few tiny petechaie in the choroid plexus, which was located slightly inferior to this target location. The locations that exhibited no broadband emissions appeared normal in histology.

### Acoustic Emissions-Based Control

**Figure 4:**
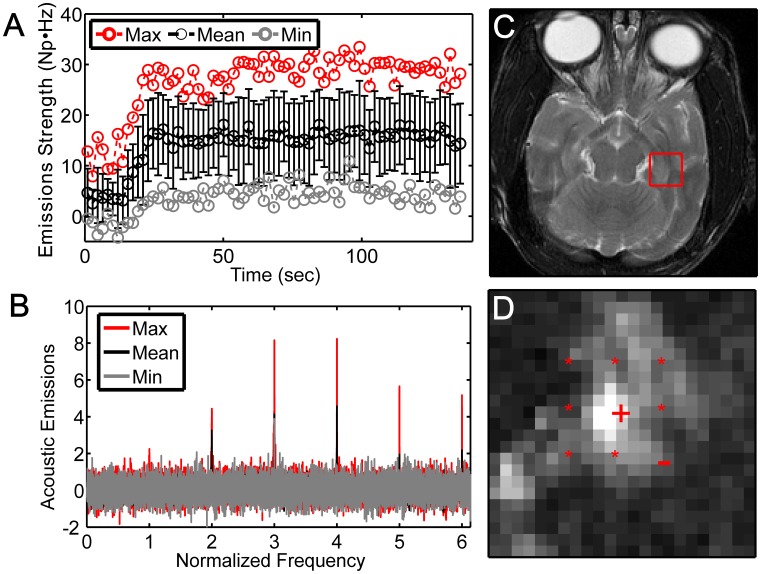
Example acoustic emissions and MRI for a multi-target sonication centered on the LGN in monkey #3. (A) Harmonic emission signal strength as a function of time (212 kPa; Mean: mean signal ± S.D. for the nine subsonications; Min./Max.: subsonications with smallest and largest signals). The increase in emissions due to the arrival of the microbubbles at about 20 s is evident. Note also the constant level of emissions over the duration of the rest of the sonication. Harmonic emissions greater than zero at time = 0 were presumably due to microbubbles present in the circulation from an earlier sonication. (B) Relative power spectra averaged between 25–140 s showing strong harmonic emissions without evident broadband emissions. (C) T2-weighted image showing the location of the sonication. (D) MRI contrast enhancement observed in T1-weighted MRI after Gd-DTPA injection (percent enhancement shown). The subsonication targets are indicated (‘+’ subsonication target with strongest signals; ‘−’ target with smallest signals; ‘*’ others).

After confirming the system performance in our pilot studies, we utilized it as a basis to manually control the acoustic power level during in a survival study in monkeys #3–6, which we performed multi-target sonications. Example acoustic emissions recordings and MRI findings from these experiments are shown in Fig. 4. During treatment, we monitored in real-time the strength of the harmonic emissions from all subsonications as a function of time (Fig. 4A). The microbubble arrival in the targeted region was apparent as a marked increase in relative harmonic emissions ∼15–20 s after the start of the infusion. After the microbubbles arrived, the signal remained at a steady level for the duration of the sonication. The spectral data from each burst were also displayed (Fig. 4B) and monitored to ensure that no broadband emission occurred. The sonication was repeated at a higher power level if strong harmonic emissions were not observed.

Overall, we found that in most cases we were able to use this control to produce MRI contrast enhancement without broadband emissions that are indicative of tissue damage. Only 3/75 (4%) of the targets of interest for the safety study that were centered on the LGN or visual cortex failed to produce detectable BBB disruption, and only 17/114 (15%) of all the multi-target sonications exhibited broadband emissions with an SNR greater than three. When broadband emissions were observed, they were typically only evident in a few of the bursts. Among the 1026 subsonication locations in these 114 multi-target sonications, over 84,000 individual bursts were applied. During the 17 sonications that produced broadband emission, only 187 bursts had broadband signal at this level. Thus, overall only about 0.2% of the bursts over these 22 sessions were delivered above the inertial cavitation threshold.

### Acoustic Emissions vs. MRI

**Figure 5:**
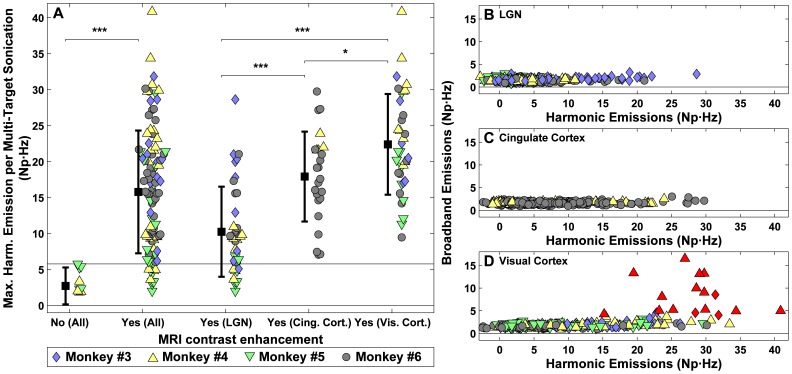
Harmonic emissions predict BBB disruption and safety limits. (A) Maximum harmonic emission signal strength achieved during 104 multi-target sonications that did and did not result in MRI contrast enhancement. Sonication at different tissue structures produced different levels of harmonic emissions (*P<0.05; ***P<0.001). The greatest harmonic emissions were measured during sonication in the visual cortex. Contrast enhancement was always observed when the harmonic signal strength was 6 Np·Hz or higher. (B–D) Broadband emission plotted as a function of harmonic signal strength for 1026 subsonication targets in the three tissue structures. Red symbols indicate subsonications where the SNR of the broadband emissions signals was greater than 3. Such emissions were only observed in sonications in the visual cortex, and with one exception, only occurred when the harmonic emissions strength was greater than 20 Np·Hz.

When data from all multi-target sonications were analyzed, we found that the strength of the harmonic emissions was predictive of whether or not MRI-evident BBB disruption occurred. This ability is evident in Fig. 5A, which plots the strength of the harmonic emissions for sonications with and without MRI contrast enhancement. The harmonic emissions were significantly higher (P<0.001) for cases where MRI contrast enhancement was detected. Ten of the multi-target sonications did not produce any evident Gd-DTPA extravasation; their harmonic emissions had a strength of 6 Np·Hz or less. Only 12/104 (11.5%) sonications where Gd-DTPA extravasation was observed exhibited harmonic emissions of less than 6 Np·Hz, which was then used as a threshold for successful BBB disruption.

The strength of the harmonic and broadband emissions was also compared (Fig. 5B–D). The risk for significant broadband emissions in the visual cortex was predicted by the strength of the harmonic emissions. With the exception of a single subsonication that overlapped a sulcus, broadband emissions appeared only when harmonic emissions signals exceeded a level of 20 Np·Hz. Thus, there was a window of harmonic signals between 6–20 Np·Hz where broadband emission was absent (i.e., “stable cavitation”); in all of these sonications successful BBB disruption was achieved. This range also agrees with the data from our pilot study (Fig. 2B). We therefore considered this range for safe exposure levels as BBB disruption is expected and inertial cavitation is unlikely.

The strength of the harmonic signals for different tissue structures varied among the different targeted structures (Fig. 5A), with sonication in the visual cortex (which always included subsonications that spanned sulci) producing the largest harmonic signals. The observed difference in harmonic emissions signals was reflected in the strength of the MRI signal enhancement after Gd-DTPA injection. Multi-target sonications centered on the LGN generally exhibited patchy and weak enhancement, while volumes in the cingulate and visual cortices produced more robust results, with substantially more enhancement occurring in gray matter regions within the volumes.

**Figure 6:**
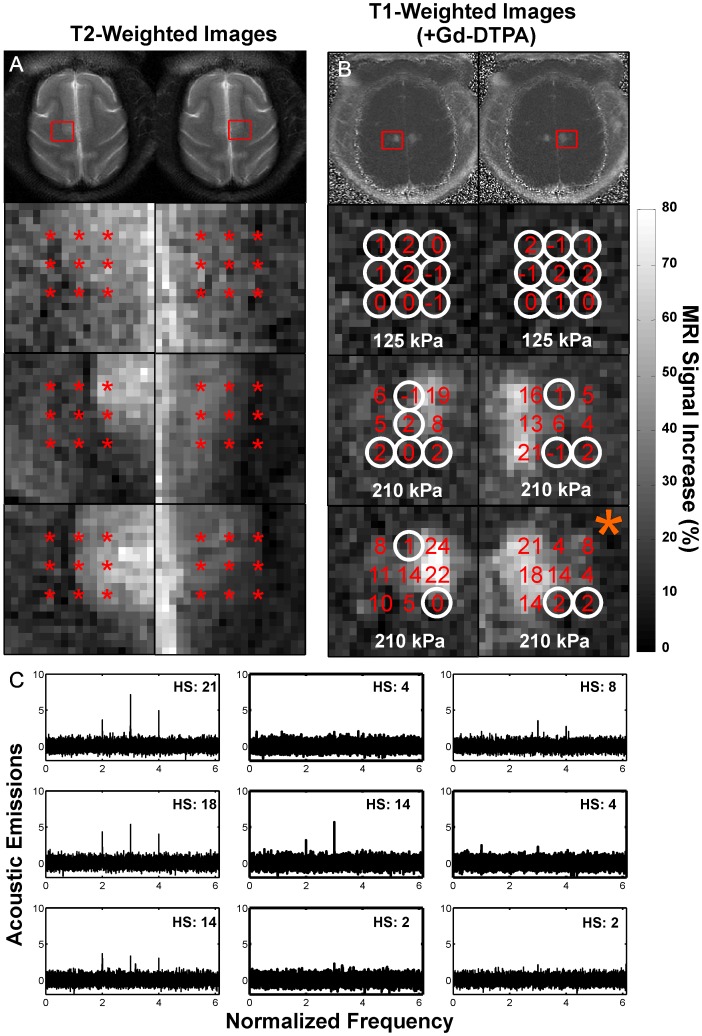
Comparison of harmonic emissions and MRI contrast enhancement at individual subsonications during six multi-target sonications in the cingulate cortex in monkey #4. (A) Axial T2-weighted FSE images showing the location of the subsonication targets, which included the cingulate cortex and adjacent white matter. Gray matter is bright compared to white matter in these images. (B) Images showing the percent increase in MRI signal after Gd-DTPA injection and the harmonic emission measurements (in Np·Hz) noted at each subsonication target. Targets where the SNR of the harmonic emissions was less than 3 (circled) did not result in detectable MRI contrast enhancement. The magnitude of the emissions agreed qualitatively with that of the contrast enhancement. Little or no contrast enhancement or harmonic emission was observed in subsonications that were targeted in white matter. (scale bar: percent MRI signal increase after Gd-DTPA injection). (C) Spectra showing only harmonic emissions at each subsonication for the multi-target sonication in (A) noted with an orange “*”. The harmonic emissions signal strength (HS) is noted for each subsonication.

We investigated this agreement in more detail by evaluating each subsonication target individually. We found that the harmonic signal was predictive of the level of signal enhancement for the different targets. Examples of this agreement are shown in Fig. 6. The magnitude of both the MRI signal enhancement and the harmonic signal at each subsonication target depended strongly on whether the target was in gray matter or white matter, where little or no extravasation or relative harmonic signal was observed. While low-level contrast enhancement was occasionally observed in voxels that appeared to be largely white matter, in most cases we could not exclude the possibility that this enhancement came from extravasation in grey matter structures partially contained within the MR imaging slice, or from leakage of Gd-DTPA from an adjacent gray matter site. In only two multi-target sonications were we confident that low-level contrast extravasation was detected in purely white matter. In both cases, the targets were sonicated two times at either an increased power level or microbubble dosage (see below).

**Figure 7:**
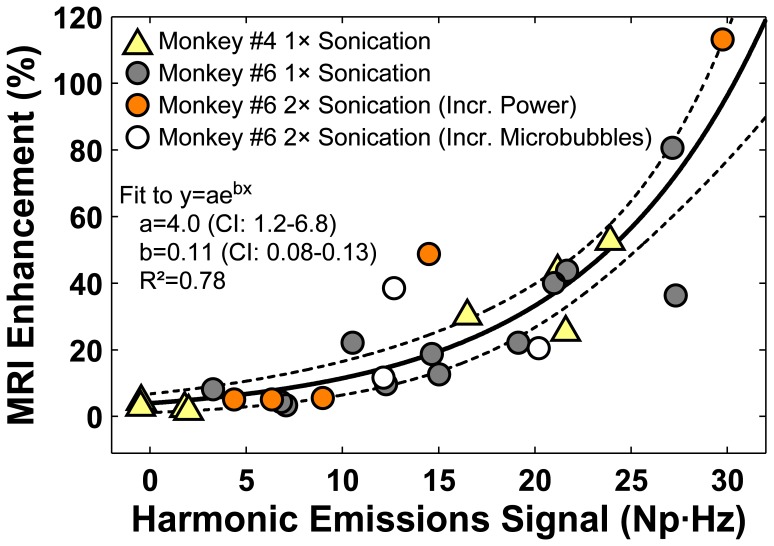
MRI signal enhancement after Gd-DTPA injection plotted as a function of the harmonic emissions signal strength. Data are shown for individual subsonication targets delivered in the cingulate cortex in monkeys #4 and #6. The MRI enhancement was found in a 3×3 voxel ROI centered on the subsonication target. The MRI signal increased nonlinearly as a function of the strength of the harmonic emission. A good correlation (R^2^: 0.78) was found in a fit of the data to an exponential (solid line; dotted lines: 95% confidence intervals). Data shown are for the subsonication target that exhibited the greatest MRI signal enhancement for each of 28 multi-target sonications that were performed in the cingulate cortex in this study and included results from experiments where a second sonication was applied at either a higher power level or with an increased microbubble dose.

In the cingulate cortex sonications, we could always associate the locations with the most MRI contrast enhancement to the corresponding subsonication target and explore the correlation between harmonic emissions quantitatively. Fig. 7 plots the percent MRI signal increase after Gd-DTPA injection for these subsonications or each of the 28 cingulate cortex sonications. Overall, the strength of the MRI signal enhancement increased non-linearly as the strength of the harmonics increased; a good correlation (R^2^ = 0.78) was observed with an exponential fit. The relationship between the strength of the harmonic emissions and the MRI enhancement level appeared to be consistent among targets in both monkeys #4 and #6 and for cases where the subsonication was targeted a second time with a higher pressure amplitude or microbubble dose (see below).

Only 5/17 (29.5%) of the targets with broadband emissions produced hypointense spots that were detected in a blind review of the T2*-weighted imaging; the rest were not found. We should note, however, that sulci always appeared hypointense in the T2*-weighted imaging. If petechaie were produced there, they would have been undetectable. The subsonications with broadband emission were all part of multi-target sonications centered on the visual cortex and occurred at 0.65 W and above (estimated pressure amplitude in the brain 180 kPa or greater). Eighty-eight percent (15/17) of the subsonication targets with broadband emission were among the three most lateral targets in the 3×3 grid and were consistent with being targeted to a large vessel-containing sulcus or a large surface vessel, suggesting that such vessels may have a lower inertial cavitation threshold.

Ultraharmonic emissions were not useful for predicting BBB disruption or damage. They were only occasionally observed (28/1026 subsonications), and when they were seen, they were detected only at exposure levels above the threshold for BBB disruption. We detected broadband emissions along with the ultraharmonic emissions in 10/28 (36%) of these subsonications. Except for four cingulate subsonications, all ultraharmonic emissions were observed during multi-target sonications centered on the visual cortex.

### Sonication Optimization

**Figure 8:**
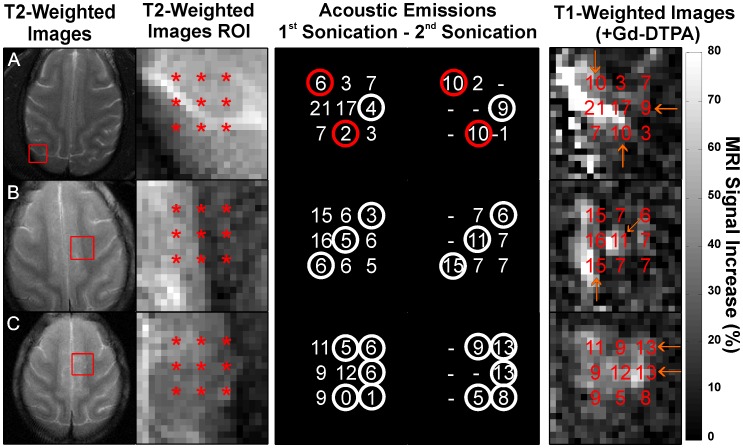
Sonication optimization. (A) Example where the pressure amplitude was increased by 10 kPa at select subsonications in a visual cortex sonication in monkey #4. The harmonic emissions achieved during the first and second sonications (in Np·Hz) are noted for each subsonication target. Two of five targets that were sonicated twice overlapped with a sulcus (red circles) and showed a large increase in harmonic emissions with the second sonication. From the other targets only one showed significant increase (circled). (B) Similar experiment performed in the cingulate cortex in monkey #5. In this example the pressure amplitude was increased by 15 kPa for the second sonication. Three of the locations showed a strong increase in harmonic emissions (circled). (C) Similar experiment performed in the cingulate cortex in monkey #5, but instead of increasing the pressure amplitude, the second sonication used five times the microbubble dose (circles indicate the most pronounced increase in harmonic emissions). Arrows indicate strong contrast enhancement at the targets with pronounced increase in the harmonic emissions (Left images: T2-weighted images showing the location of the targeted volumes and the ROI; Right images: images showing contrast enhancement in T1-weighted images after Gd-DTPA injection).

We attempted to increase the harmonic emissions (and presumably the BBB disruption) at individual subsonications that exhibited low signal by sonicating a second time at an increased power level. This procedure was tested in seven of the multi-target sonications in the cingulate or visual cortices. A total of 47/63 of the subsonications in these multi-target sonications had low harmonic emissions and were selected for a second sonication; subsonications that had strong harmonic emissions were not sonicated again. A small (5–15 kPa) increase in pressure amplitude did increase the harmonic emission in the second sonication in many of the subsonications, and that this increase was often substantial. For example, in 38 of the subsonication targets, the harmonic signal was initially less than 6 Np·Hz (where BBB disruption was not expected). We were able to increase it above this value in 14/38 of these targets, and the increase was always in the safe range defined above. While the increase in harmonic signal per kPa was variable and ranged from −0.8 to 1.5 Np·Hz, the mean value (0.20±0.37 Np·Hz) was consistent with what was observed in monkey #1 (Fig. 2B). Example data from two targets where this procedure was investigated are shown in Fig. 8A–B.

**Figure 9:**
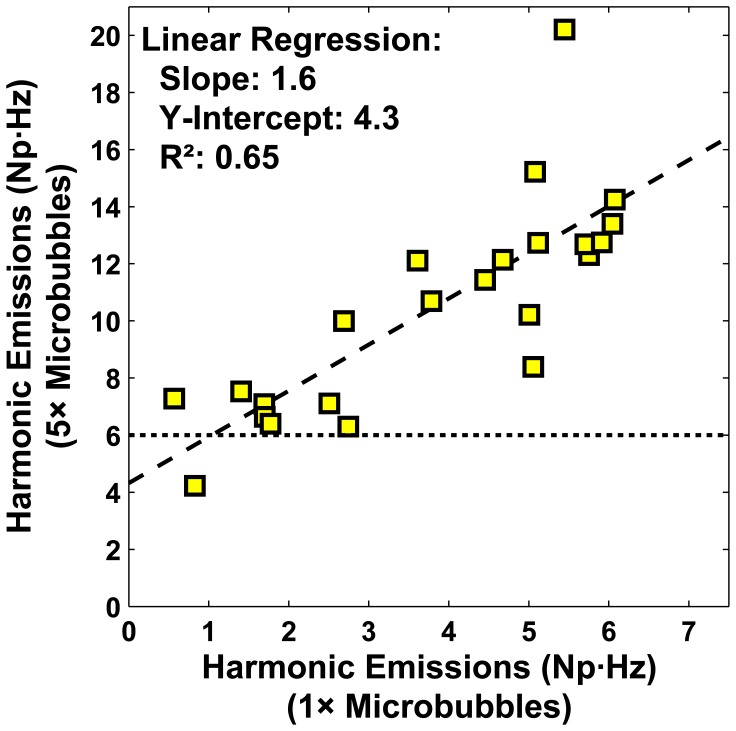
Increase in harmonic emissions strength with increased microbubble dose. Individual subsonications with low harmonic emissions signals were sonicated again with five times the microbubble dose. Signals recorded during the second sonication increased substantially; all but one increased to a level above 6 Np·Hz, a level where MRI-evident BBB disruption is expected based on results in Fig. 5. The two measurements were correlated (R^2^: 0.65). The measured slope indicates that the strength of the harmonic emissions is proportional to the number of oscillating microbubbles, since the signals were log-transformed (Eq. 3, log(5) = 1.6). A non-zero Y-intercept suggests that there may have been low-level harmonic emissions during the first sonication that were below our detection threshold.

We also investigated whether low-level harmonic emissions could be increased using a higher dose of microbubbles. This procedure was tested in four multi-target sonications in the cingulate cortex. A total of 23/36 of the subsonications had low harmonic signal and were sonicated a second time at the same power level, but with five times the micro-bubble dose. An example showing data from this procedure is shown in Fig. 8C. We found that this approach was effective at increasing the relative harmonic signal and maintaining a safe exposure based on the criteria defined above. This increase was also predictable - a linear relationship was observed between the harmonic signal for the two sonications (Fig. 9). This slope is consistent with a linear dependence of the harmonic signal strength and microbubble dose, as our measurements were log-transformed and ln(5) = 1.6. A non-zero y-intercept suggests that oscillating microbubbles were perhaps present during the first sonication, but they produced harmonics below the sensitivity of our detectors.

## Discussion

The type of microbubble oscillation that occurs during sonication can be characterized by the spectral content of passively-recorded acoustic emissions originating from the oscillating microbubbles [21]. We have demonstrated that the strength of harmonic signal is predictive of both the onset and magnitude of the MRI signal increase reflecting Gd-DTPA extravasation and are indicative of the exposure parameters needed for robust BBB permeabilization. For safe BBB disruption, broadband emissions set an upper limit on the exposure parameters, as they are a signature for inertial cavitation and tissue damage [10], [11]. These two signals collected during the sonications and the methodology to quantify them form the basis to build controllers for cavitation-based pharmacological therapies in the brain and elsewhere. Here, we showed that reliable measurements could be obtained transcranially in a relevant animal model with a clinical TcMRgFUS system, and over 22 weekly sessions demonstrated that by monitoring the emissions we could reliably ensure a safe and effective outcome. These results are highly encouraging and warrant evaluation of the method in patients.

In these experiments, the operator of the TcMRgFUS device was part of the control loop that evaluated the acoustic emissions and modified the power level for each sonication. Improved outcomes may be achieved using automated, computer-based control of the sonications [17], [42]. With such control, one could automatically adjust the exposure levels on a burst-by-burst level to rapidly achieve a desired harmonic emissions signal strength, and automatically lower or stop the sonication if broadband emissions were detected. The quality of the data recorded here, along with the correlations we observed between the emissions signals and the resulting BBB disruption suggest that this will be readily achievable. However, our data suggest that one may not be able to easily predict the response to a change in acoustic power, as we observed substantial variability among the different targets after sonicating a second time at only a slightly higher level. This variability is perhaps not surprising, as the effective volume of the focal region will increase at higher pressure amplitudes and will include different structures with different vessel densities (and microbubble concentrations). Nevertheless, using small increments in acoustic power should ensure that such control would be an effective means to ensure a safe and effective procedure. Sonicating a second time with an increased microbubble dose appeared to be a more predictable means to increase the harmonic emissions.

For this control to be translated to patients, we need to ensure that the measurements can be achieved through the thicker human skull. Our harmonic emissions data are promising for this translation. The relatively broad window in harmonic signals where safe BBB permeabilization was observed was substantially above the noise floor of our monitoring system. A harmonic emissions signal of 6 Np·Hz, the level above which where BBB disruption was always found, corresponded to an SNR of approximately 10. If we assume that a human skull is 5 mm thicker than a macaque skull, using an attenuation coefficient of 70 Np/m at 840 kHz we would expect an attenuation of the relative harmonic signals at worst by a factor of two. Even with this attenuation we could detect harmonic signals with SNR greater than five. However, measurements using a higher-frequency TcMRgFUS clinical system could be challenging since acoustic attenuation increases with frequency. In contrast to the harmonics, the broadband signals were substantially weaker, and it may be challenging to detect them in a human. For example, in our pilot study, petechaie were found in histology with a broadband signal of only 5.6 Np·Hz, (SNR = 4.3). Our acoustic emissions measurements were also not sensitive to harmonic emissions during sonication in white matter. In our previous study, we found that the BBB can be disrupted in white matter even when no harmonic emissions or MRI contrast enhancement is observed [32]. As described below, we suspect this is due to the white matter’s lower vascular density, leading to a lower microbubble concentration within the focal region. If that is the case, a more sensitive detector may be able to measure the emissions.

We anticipate that we can significantly improve the sensitivity of our measurements by optimizing the design of our detectors. For example, we expect that we could achieve a two-fold increase in sensitivity by simply aiming our receiving transducers at the targeted region, as the location of most of our sonications fell outside of the -3dB region of the sensitivity profile of the receiver transducers. Additional improvement may be achieved by using multiple detectors, or by attaching them to a positioning system to maximize the sensitivity for each target. The use of arrays of receivers and passive imaging reconstruction methods [43] to map the microbubble activity within the brain in TcMRgFUS systems [44], [45] will further improve our results. In the present study, with only two detectors we could only assume that the signals we recorded were coming from microbubble activity in the focal region. This is a reasonable assumption since we have not observed any BBB disruption or damage outside of the targeted volumes in these experiments or in our earlier work [32], but it would be desirable from a safety perspective to ensure that the activity is occurring only at the focal region.

Our measurements may also be sensitive to signals arising from nonlinear propagation due to the presence of the microbubbles in the beam path of the TcMRgFUS device [46]. While normalizing the acoustic emissions to baseline data obtained without microbubbles will remove the contributions from the transmitted and reflected wave the presence of the microbubbles can effectively change the nonlinearity of the tissue and add harmonic activity to our measurements. With the large geometric gain of the TcMRgFUS device, the relatively low microbubble concentration, and the low frequency of the TcMRgFUS device, these contributions might be expected to be small compared to emissions from the focal region. However, they should be explored. Additional uncertainties in the magnitude of the emissions arising from differences in skull thickness between subjects and from the sensitivity profile of the receiver transducers should also be taken into account.

Despite these uncertainties, we found a good correlation with the strength of the harmonic emissions to the strength of the MRI enhancement after the administration of Gd-DTPA, at least in the cingulate cortex. As the MRI contrast enhancement level after BBB disruption can be correlated to drug concentrations in the brain [47], this finding suggests that one may be able to predict the amount of drug delivered to each target without having to perform contrast-enhanced imaging. This would be desirable, since being able to perform this procedure without an MRI would reduce its costs and complexity. However, this ability might be confounded by the different responses to the sonications we observed at different brain structures. These differences were most evident in white matter, where little or no acoustic emissions or BBB permeabilization were observed, and in the visual cortex targets that included sulci, where the acoustic emissions and BBB permeabilization were substantially larger.

It is likely that this variable response, particularly in gray vs. white matter, is explained by differences in vascularity [48]. Differences in blood flow and in the interstitial space may also impact the sonications and resulting extravasation of imaging tracer or drug. It is clear that treatment planning imaging, at least, will be necessary to interpret the acoustic emissions recorded at each target. Advanced imaging methods to estimate local variations in vascularity, vessel size, blood flow, and other functional imaging methods may also prove useful in interpreting the measurements.

Differences in vessel properties may also be important with respect to safety. A lower cavitation threshold was evident for the peripheral subsonications in the visual cortex and was consistent with being targeted to a large vessel-containing sulcus or a large surface vessel. Having a lower inertial cavitation threshold in larger vessels would agree with experimental results in vessel-mimicking tunnels [49] and might occur due to higher microbubble numbers, lower bubble-to-bubble distances, or from less restrictions on the magnitude of bubble oscillations. If this is the case, it might restrict the design of TcMRgFUS systems for BBB disruption. For example, a transducer with a smaller geometric gain (i.e. not hemispherical) will have an elongated focal region, and it may not be able to target superficial regions without including vulnerable regions in the focus.

Our findings are in agreement with other studies that have evaluated acoustic emissions recordings during ultrasound-induced BBB disruption. In a prior study in rabbits [10], the presence of large harmonic emissions signals was also found to be predictive of whether or not BBB disruption occurred, and their strength was correlated with MRI signal intensity increases after Gd-DTPA administration. That work also found a correlation between broadband emissions and the production of petechaie in the brain. While we do not have histological correlation with our acoustic emissions measurements (as monkeys #3–6 were not euthanized after the sonications), we did not find changes in T2*-weighted imaging in the absence of broadband emissions. It would be interesting to examine whether the different levels of harmonic emission correlate with any changes evident in histology. Based on the results of our prior study that did include histology [32], we do not expect there to be any significant changes in the monkey brain when T2*-weighted imaging is normal. Tung et al. also observed strong harmonic emissions during BBB disruption in mice and correlated broadband emissions with histological damage [11]. That group has also shown feasibility of transcranially recording emissions in monkeys [50]. Finally, O’Reilly et al. has tested an automated, computer-controlled control method, where the acoustic power was increased sequentially until ultraharmonic emissions were detected, at which point the pressure amplitude was dropped to a lower value [17]. They found some minor petechaie in some cases, which is consistent with our findings that ultraharmonic emissions, when present, were often observed along with broadband emissions. That approach did not seem to be possible here, as ultraharmonic emissions were rarely observed outside of the visual cortex sonications.

### Conclusions

We have demonstrated that increasing the sonication exposure level until strong harmonic emissions are detected is an effective way to ensure a safe exposure level for FUS-induced BBB disruption. We also observed that the strength of these emissions is correlated with the extravasation of an MRI contrast agent that normally does not penetrate the blood-brain barrier. These results are promising for clinical translation of this technique, as the experiments were performed using a clinical TcMRgFUS device and a relevant animal model. Apart from its utility in clinical practice, it is anticipated that the method and procedure presented could be an invaluable tool for pre-clinical and neuroscience research to control the delivery of therapeutic, functional, or diagnostic compounds to brain targets at a desired concentration. Overall, the present work describes a basis for “smart therapeutic ultrasound systems” that can control bubble dynamics in real-time and in vivo to obtain reproducible, safe, and uniform permeabilization of vascular barriers in the brain, and potentially elsewhere. Based on the presented data and analysis the next step is to develop a fully automatic feedback controller of the sonication exposure settings. Such systems are expected to be an important methodology to asses bubble kinetics and provide treatment control. This control is anticipated to be especially important at diseased targets such as tumors, where local anomalies in vascularity and flow are frequent.
